# Methodological Clarification and Analysis of Demographic and Anthropometric Determinants in the Calculation of REMS Bone Mineral Density

**DOI:** 10.1007/s00223-026-01547-1

**Published:** 2026-05-19

**Authors:** Francesco Conversano, Paola Pisani, Sergio Casciaro

**Affiliations:** 1 Institute of Clinical Physiology, National Research Council, c/o Campus Ecotekne (Ed. A7), Via Prov.Le Lecce-Monteroni, 73100 Lecce, Italy; 2 Echolight SpA, Lecce, Italy

**Keywords:** Radiofrequency echographic multi spectrometry, REMS, Bone mineral density, Femoral neck, Total hip, Lumbar spine

## Abstract

Radiofrequency echographic multi spectrometry (REMS) derives bone mineral density (BMD) using age- and anthropometry-dependent spectral models and conversion equations. The aim of this paper was to quantify the fraction of REMS BMD that can be explained by demographic and anthropometric variables. We analyzed 8000 proximal-femur and 8000 lumbar-spine REMS scans from Caucasian women, with independent development pools and fixed test sets of 4000 scans each and no significant development-versus-test differences in age, anthropometry, or REMS BMD. In 100 repeated runs, linear regression equations were fitted on random training subsets of 100–400 development scans. The primary anthropometric model equation included age and weight only. A further hypothetical maximum-explainability expanded model additionally included height, squared terms, and pairwise interactions, to explore the upper bound of explainability obtainable from anthropometric inputs alone. At train size 400, primary-model train/test R^2^ values were 0.77/0.58 at the lumbar spine, 0.83/0.57 at the total hip, and 0.93/0.70 at the femoral neck; corresponding expanded-model values were 0.84/0.66, 0.89/0.69, and 0.95/0.75, respectively. Thus, although an association between REMS BMD and demographic/anthropometric variables was expected by design and resulted relevant even under independent testing, the primary model left 30–43% of REMS BMD unexplained by age and weight. The expanded model increased test R^2^, but still left a substantial fraction of REMS BMD unexplained by anthropometric inputs. The highest R^2^ values were consistently observed only in training data. Additional sensitivity analyses using alternative anthropometric models yielded similar findings. In conclusion, anthropometric explainability of REMS BMD was strongly sample-dependent and systematically much lower under independent testing than suggested by same-cohort analyses.

**Supplementary Information:**

The online version contains supplementary material available at 10.1007/s00223-026-01547-1.

## Introduction

Radiofrequency echographic multispectrometry (REMS) is a non-ionizing technology for axial skeletal assessment at lumbar spine and proximal femur. In standard clinical use, raw radiofrequency (RF) ultrasound signals acquired at the target site are processed to generate patient-specific spectra, which are compared with reference spectral models stratified by anatomical site and by patient characteristics such as age, sex, and body mass index (BMI).The resulting similarity score (named Osteoporosis Score) is then converted into bone mineral density (BMD) through stratum-specific equations [1–3]. Therefore, sex, age, and body habitus select the reference context against which patient-specific spectral information is interpreted. Validation studies have reported good agreement with dual-energy X-ray absorptiometry (DXA), as well as clinically relevant diagnostic performance, also in large multicenter cohorts [1–6].

Since REMS algorithm is designed to operate within an anthropometrically matched reference framework [2, 3], some degree of association between REMS BMD and demographic/anthropometric variables is expected by design. It may plausibly be stronger than that observed for DXA, which is also significantly associated with age, weight, and BMI in general populations [7–10]. The clinically and methodologically relevant question is therefore not whether REMS BMD is associated with demographic or anthropometric variables, but whether these variables are sufficient to determine REMS BMD to such an extent that patient-specific spectral information would become marginal.

Formal quantification of the actual influence of anthropometric and demographic features on REMS BMD calculation is an interesting topic that, until now, has been explored only in a very preliminary way. It has been performed on small cohorts, employing single-run analysis and same-cohort fitting and evaluation in the place of an independent test set, providing highly heterogeneous results. In these small studies age and body habitus apparently explain a percentage of REMS BMD variability ranging from 56% to over 90% [11, 12].

Anthropometric equations predicting DXA-derived BMD have also been reported to achieve apparently high R^2^ values (up to 0.90) in pediatric, athletic, or convenience samples. However, these studies typically relied on same-sample fitting, lacked a large independent test set, or investigated unusually homogeneous populations [13–15]. Such designs can falsely amplify the apparent determinism of demographic and anthropometric models for BMD, independently from REMS.

The present paper addresses the quantification of the dependence of REMS BMD on demographic and anthropometric variables, aiming in particular at determining the actual residual explainability when development and test sets are fully independent yet explicitly matched and performance is evaluated in a fixed large test set over repeated random extractions from the development set rather than from a single run.

## Methods

### Study Design and Data Source

This is a retrospective methodological secondary analysis performed on 16,000 de-identified REMS scans retrieved from the manufacturer database and acquired between 2015 and 2024. The database includes only de-identified data previously acquired in different clinical settings, including routine clinical use, previous clinical studies, national and international multicentric validation cohorts, post-market surveillance. Anyway, data acquisition was always fully independent of the present paper analyses. The analysis was focused on Caucasian women only and included both lumbar and femoral scans. Each included scan corresponded to a different patient. All the available scans were reprocessed with the latest available version of EchoStudio software (v2.3.0, Echolight SpA, Lecce, Italy).

At the proximal femur, both femoral-neck (FN) and total-hip (TH) REMS-BMD outputs were generated from the same 8000 scans, meaning the FN and TH analyses were based on exactly the same scans and on the same development/test partition (see next paragraph). A separate set of 8000 lumbar-spine (LS) scans was used for the spine analyses.

### Study Cohort Construction

For each scan type (lumbar or femoral), the study cohort was constructed according to three pre-specified criteria.

First, only scans fulfilling the highest quality requirements as reported in the EchoStudio User Manual were eligible, in order to minimize acquisition-related noise and ensure that the analysis addressed the actual dependence of REMS outputs rather than the consequences of poor scans. At the proximal femur, eligible scans had to display the longitudinal proximal-femur profile (head, neck, and trochanter), with the femoral-neck interface centered in the B-mode image, parallel to the focus line and just below it after correct depth and focus setting. At the lumbar spine, vertebral interfaces had to be centered in the image, parallel and close to the focus line, and below it. In both sites, scans showing poor probe-skin coupling, inadequate depth/focus selection, nonrepresentative bone-profile positioning, excessive interface inclination (greater than 10°), or a sub-optimal number of valid frames (less than 80%) were not eligible.

Second, the minimum training-sample size was fixed at 100 scans, and a total of four increasing training-set sizes were evaluated (100, 200, 300, and 400 scans), with the requirement that both the development pool and the fixed test set should remain independent and at least ten times larger than the corresponding training subset. This requirement defined the amount of high-quality scans needed for the final design: 8000 per anatomical site.

Third, two independent sets of 4000 cases each were randomly extracted for each site from the whole batch of the scans fulfilling the above-mentioned quality requirements: the first was labelled as “development pool” and the second as “fixed test set” and both underwent dedicated statistical testing to verify the absence of significant differences in demographic, anthropometric, and site-specific REMS BMD distributions. This matching step was deliberately conservative: by reducing anthropometric and densitometric differences between development and test, it tends to favor rather than penalize the apparent generalizability of anthropometric equations.

Therefore, the fixed test set was independent from the development pool in the sense that no scan used for model fitting was included in the test set, but it should be regarded as an internal independent test set derived from the same overall data source, not as an external validation cohort.

### Anthropometric Regression Models

In the context of this paper, “anthropometric models” refers to multiple linear regression equations fitted on experimental REMS data with REMS BMD as the dependent variable and demographic/anthropometric variables as predictors. Separate equations were derived for LS, TH, and FN.

The primary anthropometric model included only age and weight as predictors of REMS BMD. This model was chosen because it captures the most clinically relevant claim emerging from recent literature [11]. The main quantitative result of the present study is the fraction of REMS BMD not explained by this primary model under independent testing.

A secondary hypothetical maximum-explainability expanded model was also considered as a deliberately permissive anthropometric specification, intended to test whether adding height, squared terms and pairwise interactions would significantly increase explainability. This model should not be interpreted as a definitive mathematical upper bound of all possible anthropometric effects, but as a conservative stress test of the primary “age + weight” findings.

Two additional sensitivity models were also evaluated: an “age + BMI” model and an “age + weight + BMI” model.

### Repeated Random Resampling and Performance Metrics

For each site and each model, 100 random training subsets of size 100, 200, 300, and 400 were drawn without replacement from the development pool. A new regression equation was fitted in each run and then applied to the same fixed independent test set of 4000 scans. In the context of this paper, “in-sample” performance denotes performance on the training subset used to fit a given equation, whereas “out-of-sample” performance denotes performance on the fixed independent test set.

Performance was summarized by the coefficient of determination (R^2^). For each train size and model, median values across the 100 runs were reported together with the full min–max range. Unexplained test variance was calculated as 100 × (1 − test R^2^).

### Statistical Analysis

Continuous variables are reported as mean ± standard deviation (SD) or, where specified, as ranges derived from repeated resampling. Development-versus-test comparability was assessed with Welch’s t-test for age, height, weight, BMI, and site-specific REMS BMD, and further described with standardized mean differences (SMDs) and overlap coefficients derived from kernel-density estimation (OVL-KDE). Low absolute SMDs and OVL-KDE values close to 1 indicate strong distribution overlap. Primary analyses used ordinary least-squares linear regression equations fitted on random development subsets and evaluated on fixed independent test sets. Exploratory sensitivity analyses additionally examined two BMI-containing models (“age + BMI” and “age + weight + BMI”), ridge- and lasso-penalized versions of the hypothetical model, and a spline-based model. The “age + weight + BMI” model was treated as a supplementary sensitivity specification because BMI partly recapitulates information already carried by weight; variance inflation factors (VIFs) were therefore calculated to quantify that redundancy. Penalized models were included because they shrink unstable coefficients and can therefore reveal whether residual variance unexplained by anthropometric inputs mainly reflects overfitting, whereas the spline-based model was included because it allows smooth curved relationships without forcing a fixed quadratic shape. Adjusted R^2^ was examined for unpenalized models as a supplementary descriptor only, whereas generalizability was judged primarily from fixed-test R^2^. To formally evaluate the consistency of the train-versus-test R^2^ gap, paired Wilcoxon signed-rank tests were performed across the 100 repeated runs at train size 400, comparing train R^2^ and fixed-test R^2^ within each site and model family. In addition, 10th–90th percentile intervals were calculated from the same run-level outputs used to generate the median and min–max summaries. Statistical significance was set at *p* < 0.05. All data analyses were performed using MATLAB R2025b (MathWorks, Natick, MA, USA).

## Results

### Study Cohort

The final lumbar-spine cohort had mean (± SD) age 60.3 ± 12.0 years and mean (± SD) BMI 24.5 ± 3.6 kg/m^2^, and the final proximal-femur cohort had mean (± SD) age 60.6 ± 13.1 years and mean (± SD) BMI 24.5 ± 4.0 kg/m^2^. Both were broadly comparable with the population undergoing lumbar spine scans in the largest available prospective study on REMS technology (LS median age 60 years; LS median BMI 24.1 kg/m^2^; proximal femur median age 61 years and median BMI 24.5 kg/m^2^) [4].

No significant development-versus-test differences were detected for any variable at either site (Tables 1 and 2). In addition to what shown in Tables 1 and 2, in the lumbar cohort absolute SMDs did not exceed 0.013 and minimum OVL-KDE was 0.964 (Supplementary Table S1). Similarly, in the proximal femur cohort, absolute SMDs did not exceed 0.025 and minimum OVL was 0.945 (Supplementary Table S1). Together, these metrics indicate substantial practical overlap between development pools and corresponding fixed independent test sets.

**Table 1:** Final lumbar-spine analytical cohort: comparison between development pool and fixed independent test set (Values are reported as mean ± SD)

Variable	Overall cohort (N = 8000)	Development pool (N = 4000)	Fixed test set (N = 4000)	*p*
Age, y	60.3 ± 12.0	60.3 ± 11.9	60.3 ± 12.2	0.98
Height, cm	163.8 ± 8.1	163.8 ± 8.0	163.8 ± 8.1	0.93
Weight, kg	65.9 ± 11.0	65.9 ± 10.9	65.8 ± 11.1	0.72
BMI, kg/m^2^	24.5 ± 3.6	24.5 ± 3.6	24.5 ± 3.6	0.65
LS REMS BMD, g/cm^2^	0.856 ± 0.109	0.855 ± 0.105	0.857 ± 0.113	0.55

**Table 2:** Final proximal-femur analytical cohort: comparison between development pool and fixed independent test set (Values are reported as mean ± SD)

Variable	Overall cohort (N = 8000)	Development pool (N = 4000)	Fixed test set (N = 4000)	*p*
Age, y	60.6 ± 13.1	60.5 ± 13.1	60.7 ± 13.1	0.66
Height, cm	164.1 ± 8.7	164.1 ± 8.4	164.1 ± 9.1	0.82
Weight, kg	66.0 ± 11.8	66.2 ± 12.3	65.9 ± 11.4	0.26
BMI, kg/m^2^	24.5 ± 4.0	24.5 ± 4.0	24.5 ± 3.9	0.56
TH BMD, g/cm^2^	0.778 ± 0.135	0.777 ± 0.132	0.779 ± 0.138	0.41
FN BMD, g/cm^2^	0.643 ± 0.112	0.644 ± 0.110	0.642 ± 0.114	0.43

### Primary Anthropometric Model (Age + Weight)

**Fig. 1:**
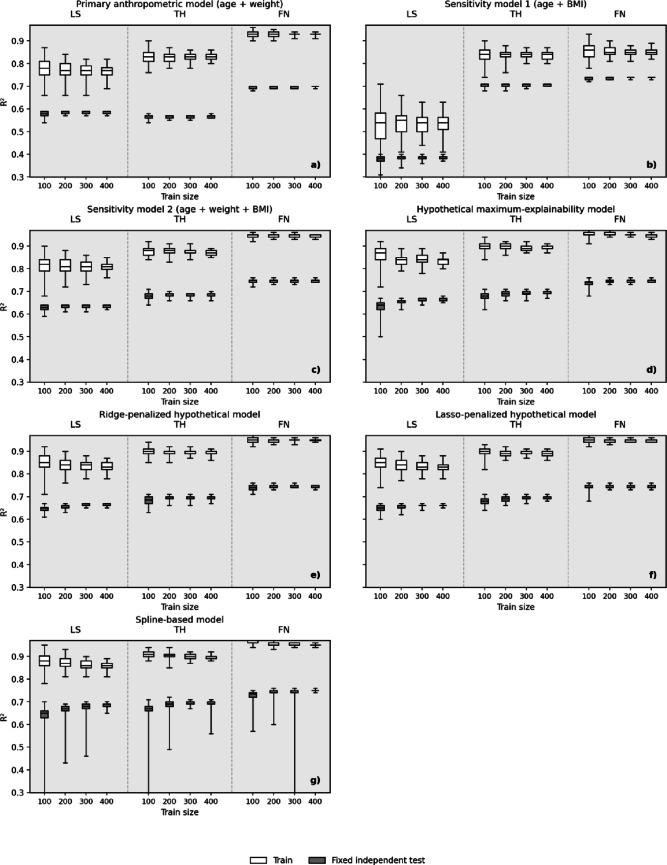
Box plots of train and fixed independent test R^2^ across 100 repeated random extractions for different model families: **a** primary anthropometric model (age + weight), **b** sensitivity model 1 (age + BMI), **c** sensitivity model 2 (age + weight + BMI), **d** hypothetical maximum-explainability model, **e** ridge-penalized hypothetical model, **f** lasso-penalized hypothetical model, and **g** spline-based model. Each panel shows lumbar spine (LS), total hip (TH), and femoral neck (FN) blocks, with dedicated box plots for each considered train sizes. Each boxplot reports the median (central line), interquartile range (box), and minimum-to-maximum range (whiskers) across the 100 repeated runs. In panel (**g**) some lower spline-test whiskers extend below the displayed range: these isolated extreme whiskers reflect a very small number of values in which prediction error exceeded the variance of the evaluation set (see the text for further details)

In the primary anthropometric model, in-sample performance was consistently higher than independent-test performance at all sites (Table 3; Fig. 1). At train size 400, median train/test R^2^ values were 0.77/0.58 at LS, 0.83/0.57 at TH, and 0.93/0.70 at FN. The corresponding independent-test variance unexplained by age and weight was 42% at LS, 43% at TH, and 30% at FN. Conversely, age and weight captured a non-trivial fraction of REMS BMD variability also in the fixed test set, as expected from the REMS reference framework. However, the systematic train-test gap indicates that this association should not be interpreted as full determinism of REMS BMD output.

**Table 3:** Primary anthropometric model (age + weight): median performance across 100 repeated random extractions

Site	Train size	Train R^2^ [min–max]	Test R^2^ [min–max]	Unexplained test variance (%)
Lumbar spine	100	0.78 [0.66–0.87]	0.58 [0.54–0.59]	42
	200	0.77 [0.66–0.84]	0.58 [0.57–0.59]	42
	300	0.77 [0.66–0.82]	0.59 [0.57–0.59]	41
	400	0.77 [0.69–0.82]	0.58 [0.57–0.59]	42
Total hip	100	0.83 [0.76–0.90]	0.57 [0.54–0.58]	43
	200	0.83 [0.78–0.87]	0.57 [0.55–0.57]	43
	300	0.83 [0.78–0.86]	0.57 [0.55–0.57]	43
	400	0.83 [0.80–0.86]	0.57 [0.56–0.58]	43
Femoral neck	100	0.93 [0.90–0.96]	0.69 [0.68–0.70]	31
	200	0.93 [0.90–0.95]	0.70 [0.69–0.70]	30
	300	0.93 [0.91–0.94]	0.70 [0.69–0.70]	30
	400	0.93 [0.91–0.94]	0.70 [0.69–0.70]	30

At the lumbar spine, across train sizes 100–400, median train R^2^ was essentially stable at 0.77–0.78, although showing significant run-to-run variability, especially for the lowest train sizes (maximum range 0.66–0.87). Median test R^2^ was also essentially stable at significantly lower values (0.58–0.59), with limited run-to-run variability (Table 3; Fig. 1). Accordingly, 41–42% of LS REMS-BMD variance remained unexplained by age and weight in the independent fixed test set, even under the favorable condition of matched-cohort setting.

Total hip results were similar to those of the lumbar spine, although presenting a slightly higher and constant value of median train R2 (0.83) (Table 3; Fig. 1). However, maximum test R2 was never higher than 0.58, indicating that under no circumstances age and weight alone could explain more than 58% of TH REMS-BMD variance.

At the femoral neck, the primary model showed higher in-sample performance (median train R2 stable at 0.93), but this dropped in the independent test setting (median test R2 essentially stable at 0.69–0.70) (Table 3; Fig. 1). Furthermore, the reported constant value of maximum test R2 (0.70), indicated that, across the whole considered range of train sizes, at least 30% of FN REMS-BMD variance could never be explained by age and weight alone.

### Secondary Hypothetical Maximum-Explainability Model

The secondary model increased train R^2^ at all sites, but the gain under independent testing was more modest (Table 4; Fig. 1). At train size 400, median train/test R^2^ values were 0.84/0.66 at LS, 0.89/0.69 at TH, and 0.95/0.75 at FN. The corresponding independent-test variances unexplained by anthropometric inputs were 34%, 31%, and 25%. Thus, even when the regression equations were expanded with terms lacking a clear biological relevance that might conceivably influence a physical interpretation, a substantial portion of REMS BMD still remained unexplained by anthropometric inputs under independent testing.

**Table 4:** Secondary expanded model (age, weight, height, squared terms, and pairwise interactions): median performance across 100 repeated random extractions

Site	Train size	Train R^2^ [min–max]	Test R^2^ [min–max]	Unexplained test variance (%)
Lumbar spine	100	0.87 [0.72–0.92]	0.64 [0.50–0.67]	36
	200	0.84 [0.79–0.89]	0.66 [0.62–0.67]	34
	300	0.84 [0.78–0.89]	0.66 [0.64–0.67]	34
	400	0.84 [0.80–0.87]	0.66 [0.65–0.68]	34
Total hip	100	0.90 [0.84–0.94]	0.68 [0.62–0.71]	32
	200	0.90 [0.86–0.92]	0.69 [0.66–0.71]	31
	300	0.89 [0.87–0.92]	0.69 [0.66–0.71]	31
	400	0.89 [0.87–0.91]	0.69 [0.67–0.71]	31
Femoral neck	100	0.95 [0.91–0.97]	0.74 [0.68–0.76]	26
	200	0.95 [0.94–0.97]	0.75 [0.73–0.76]	25
	300	0.95 [0.94–0.96]	0.75 [0.73–0.76]	25
	400	0.95 [0.93–0.96]	0.75 [0.74–0.76]	25

### Additional Model Families and Robustness Analyses

Supplementary BMI-containing sensitivity analyses and exploratory flexible-model analyses are summarized in Fig. 1 and Supplementary Table S2. At train size 400, the “age + BMI” model yielded median train/test R^2^ values of 0.54/0.39 at the lumbar spine, 0.84/0.71 at the total hip, and 0.85/0.74 at the femoral neck. The corresponding “age + weight + BMI” model yielded 0.81/0.64, 0.87/0.68, and 0.94/0.75, respectively.

The VIFs of the “age + weight + BMI” model remained moderate rather than extreme (lumbar spine: age 1.12, weight 2.99, BMI 3.06; proximal femur: age 1.11, weight 3.51, BMI 3.58), as detailed in Supplementary Table S3, indicating some redundancy between weight and BMI but not the degree of numerical instability that would invalidate the fit. At train size 400, ridge-penalized versions of the hypothetical model yielded median test R^2^ values of 0.66 at the lumbar spine, 0.69 at the total hip, and 0.75 at the femoral neck; the corresponding lasso values were 0.66, 0.69, and 0.75, and the spline-based model yielded 0.68, 0.70, and 0.75. In practical terms, shrinking the coefficients or allowing smoother curved anthropometric relationships changed independent-test performance in a very modest or negligible manner, with the spline-based model increasing median test R^2^ by at most 0.02 compared with the expanded model at train size 400. Supplementary run-level summaries showed that, at train size 400, train R^2^ was higher than fixed-test R^2^ in every repeated run across all sites and model families (Supplementary Tables S2).

Adjusted R^2^ values for unpenalized models at train size 400 are also reported in Supplementary Table S2. They were very close to ordinary R^2^ values and did not modify the interpretation of the comparison between primary, BMI-containing, and expanded models. At train size 400, train R^2^ was higher than fixed-test R^2^ in every repeated run across all sites and model families. Paired Wilcoxon signed-rank tests confirmed the consistency of this train-versus-test gap for all comparisons (all *p* < 0.001; Supplementary Table S4).

In a small number of spline-based runs, fixed-test R^2^ values were negative. A negative test R^2^ indicates that the fitted model predicted the fixed test-set outcomes worse than a null model based only on the test-set mean. These isolated values are consistent with instability of flexible spline fits in some resampling conditions and support the interpretation that allowing smoother nonlinear anthropometric relationships did not materially increase generalizable explainability. Median and 10th–90th percentile intervals for train and fixed-test R^2^ across all model families and train sizes are reported in Supplementary Table S5, complementing the min–max ranges shown in the main tables and Fig. 1.

## Discussion

This paper examined the fraction of REMS BMD that can be explained by demographic and anthropometric variables when regression equations are repeatedly derived on random subsets of a large development pool and then evaluated on a fixed independent test set. The key result is that, although demographic and anthropometric variables are expected to be associated with REMS BMD and can show very high apparent explainability in training data (up to 95% of the variance), their performance systematically decreased under independent testing, leaving up to 43% of REMS BMD variance not explainable from demographic and anthropometric variables.

The most relevant results are those obtained through the primary anthropometric model, because it directly tests the “age + weight” claim emerging from recent literature. At the maximum considered train size (400 scans × 100 random repetitions), 42% of LS REMS-BMD variance, 43% of TH REMS-BMD variance and 30% of FN REMS-BMD variance remained unexplained by equations based on age and weight when tested on the independent test set of 4000 scans. At the same time, the same equations generated very strong apparent explainability (up to 94%) when evaluated on the same data used to derive them (Table 3). This does not imply that age and weight have a negligible role, but indicates that their role may be substantially overestimated when model fitting and model evaluation are performed in the same cohort.

Importantly, our development and fixed test sets were purposedly constructed to be highly comparable in age, anthropometry, and BMD. That choice makes the analysis conservative, because it increases the apparent anthropometric dependence. In routine clinical use, external populations may be less matched than our fixed test set, so real-world generalization of anthropometric model performance in predicting REMS BMD could easily be even worse than what has been shown in the present analysis.

In this context, and with the purpose of conservatively challenging the primary findings, we also considered a secondary hypothetical maximum-explainability expanded model. By adding height, squared terms, and pairwise interactions, this model increased both train R^2^ and test R^2^, but still left at least one fourth of FN REMS-BMD variance and roughly one third of TH and LS REMS-BMD variance unexplained by anthropometric inputs under independent testing. Moreover, several of its terms are difficult to justify in biological or physical terms. Therefore, the secondary model should be interpreted as a deliberately permissive exploratory stress test rather than as a clinically meaningful biological model of bone physiology or definitive mathematical upper bound. The residual variance unexplained by anthropometric inputs is compatible with a contribution from patient-specific RF signal content and skeletal information carried by the REMS ultrasound spectra, although it may also include unmeasured clinical, acquisition-related, technical, or algorithmic factors not captured by the anthropometric models tested here.

Supplementary BMI-containing models do not alter this interpretation. The “age + BMI” equation behaved very differently across sites, performing poorly at the lumbar spine but better at the hip, but this does not contradict the broader message of the present work. In fact, BMI, which does not necessarily capture site-specific anatomical and acoustic conditions in the same way at spine and hip, may act at the total hip as a compact descriptor of body habitus that captures the relevant anthropometric pattern relevant to REMS acquisition more directly than weight and height entered separately. On the other hand, at the lumbar spine this compact descriptor may be less informative than weight and height considered separately or in combination with other terms. This site dependence reinforces the need to avoid extrapolating anthropometric explainability from one skeletal site to another. Accordingly, a simpler equation can occasionally generalize slightly better than a more complex one without changing the broader conclusion that a substantial fraction of REMS-derived BMD remains unexplained by anthropometric inputs under independent testing. In simple terms, adding BMI to age and weight produced only limited gains, keeping at least one fourth of REMS BMD variance always unexplained by anthropometric and demographic inputs.

The exploratory ridge and lasso models were included because they reduce coefficient instability and therefore help assess whether the remaining variance unexplained by anthropometric inputs mainly reflects overfitting rather than a true limit of anthropometric predictors. The spline-based model was included because it allows smooth curved anthropometric relationships without forcing a fixed quadratic form. In practice, neither strategy materially changed independent-test R^2^. This indicates that the unexplained component is not simply an artifact of coefficient instability or of having chosen an overly rigid polynomial equation, but more likely reflects a portion of REMS-BMD variability that is not captured by anthropometric inputs alone in this setting.

The present findings also help to contextualize the high same-cohort R^2^ values recently reported by Chan et al. [11]. Their work showed that entered age and weight influence REMS outputs, but the regression analysis was performed in a relatively small cohort and assessed on the same cohort used for model fitting. Our data suggest that such design can overstate anthropometric/demographic explainability and, importantly, its generalizability. In the present study, if we consider the most “anthropometrically-explainable” site, which is FN, the primary anthropometric model equation could achieve train R^2^ values around 0.93, which is compatible with the results reported by Chan et al. [11], but independent-test R^2^ never goes beyond 0.70. Thus, even the strongest apparent anthropometric explainability was concentrated in the model-fitting conditions rather than preserved under independent evaluation. This is a methodological clarification, not a denial that anthropometric inputs matter. Therefore, our findings should not be read as contradicting the presence of anthropometric effects on REMS outputs, but as showing that the magnitude and generalizability of those effects require independent testing.

The broader REMS literature is also consistent with this interpretation. In systemic lupus erythematosus (SLE) patients, Diz Lopes et al. [12] reported R^2^ values of 0.57 at LS, 0.70 at FN, and 0.74 at TH for REMS BMD predicted by age, sex, and BMI, using the same data for both fitting the equations and assessing their effectiveness. Their estimates, especially for FN and TH, are more coherent with our independent-test results than what would be expected for “in-sample” performance. This is likely due to the specific disease condition of the considered patients and confirms that the apparent performance of anthropometric models in predicting REMS BMD is expected to significantly decrease when non-matched populations are considered. For instance, the “age + BMI” model equations used in the present study for TH REMS-BMD estimation, which were derived from 100 fitting procedures on 400-scan subsets of the development pool and showed a maximum explainability performance of 71%, would likely have a much lower performance if tested on the patients studied by Diz Lopes et al. [12], given that the performance of an equation fitted on the same data was already slightly lower (70%). The same study [12] also showed that both REMS and DXA distinguished patients with vertebral fractures from those without vertebral fractures across the examined skeletal sites with similar fracture-discrimination performance, further confirming that the reported correlation levels between anthropometric/demographic variables and REMS BMD do not affect REMS diagnostic effectiveness in clinical contexts.

Longitudinal REMS studies support the view that anthropometric inputs are not sufficient to explain the observed outputs. Ramirez Zegarra et al. [16] documented a significant decline of about 1.9% in femoral neck REMS BMD during pregnancy and found no independent association between the measured change and maternal demographic variables. Likewise, Arechavaleta-Velasco et al. [17] reported significant gestational decline in REMS BMD together with marked interindividual variability, to the point that women could be stratified into bone-loss and bone-gain patterns despite belonging to the same general physiological setting. In women treated with romosozumab, Semeraro et al. [18] reported 6-month gains in REMS BMD of + 3.7% at TH and + 4.1% at FN while weight remained stable. In type 2 diabetes mellitus (T2DM) patients, Al Refaie et al. [19] showed that patients with higher weight and BMI than controls did not have systematically higher REMS BMD, which, contrary to what could be expected on the basis of anthropometric predictions, was significantly lower at both LS and TH and numerically lower at FN. Taken together, these observations are difficult to reconcile with a model in which REMS-BMD is systematically determined by age and body size alone.

Overall, all possible data on anthropometric/demographic explainability of REMS BMD deserve a cautious reading. Since the REMS algorithm intentionally incorporates demographic and anthropometric data, correlations with age and weight are unsurprising. What matters is whether those demographic and anthropometric inputs are sufficient to explain the REMS output. Our reported results indicate that those inputs are not sufficient: once a rigorous, independent testing is performed, even if regression equations are fitted 100 times on 400 random scans, even if the test set was conservatively matched to the development pool, and even after additional physiologically-meaningless anthropometric terms were introduced, a substantial portion of REMS BMD variance remained unexplained by anthropometric and demographic variables for each of the three considered anatomical sites: 34% for LS, 31% for TH and 25% for FN (Table 4).

The DXA literature provides a further instructive parallel. Reid et al. [7] found that age and body composition explained a substantial fraction of total and regional DXA-BMD variance in normal postmenopausal women. Morin et al. [8] reported that weight and BMI predicted DXA BMD and fractures in women aged 40–59 years. Several further studies have proposed anthropometric equations to estimate DXA BMD: Gomez-Campos et al. [13] reported DXA-BMD prediction models with R^2^ values of 0.79 in boys and 0.76 in girls; in young soccer players, Gomez-Campos et al. [14] reported high same-cohort explained DXA-BMD variance (up to 90%) in a highly homogeneous athletic sample; Aflatooni et al. [15] described anthropometric models for estimation of DXA BMD in adult subjects reporting R^2^ values up to 0.89. However, the absence of widespread clinical replacement of DXA by such equations is itself informative: high same-cohort anthropometric predictability does not make imaging redundant, especially when independent validation is limited or absent or considered populations are unusually homogeneous.

The present paper has several strengths. Firstly, both the proximal-femur and lumbar-spine cohorts contained 8000 high-quality scans, each subdivided into a development pool and an independent test set of 4000 scans each, exceeding the size of the population reported in the largest available European multicenter prospective study [4]. Secondly, the final development pools and fixed test sets were explicitly balanced on demographic, anthropometric, and BMD variables. Thirdly, the repeated-sampling design avoided overinterpreting a single favorable run, and the fully independent fixed test set provided a highly reliable estimate of what anthropometric equations retain beyond their derivation sample.

The conducted analysis has also limitations. It was retrospective, with the corresponding intrinsic limitations of retrospective analyses, although this was unavoidable in order to achieve in a timely fashion the very significant numbers required for robust statistical analysis and effective re-sampling. Although the development/test partition, repeated-resampling framework, and statistical analyses were objective and reproducible, potential selection or interpretation bias cannot be completely excluded. The fixed test set was independent from the development pool but was derived from the same overall data source, therefore the present analysis should be regarded as an internal independent test rather than as an external validation study. Only Caucasian women were analyzed, which limits generalizability to men and other ethnic groups. No head-to-head DXA comparison was included, since the purpose was not to reassess REMS accuracy against DXA, already addressed in several published validation studies, but to specifically quantify demographic/anthropometric explainability of REMS BMD. Consequently, the present study does not determine whether REMS-DXA differences, diagnostic classification, or fracture-prediction performance are influenced by age, weight, or BMI. Finally, in order to provide the most reliable estimates of the role of anthropometric and demographic determinants, only high quality REMS scans were considered (as defined by standard manufacturer’s specifications): the effect of lower scan quality on the performance of anthropometric models, as well as the analysis replication using data not sourced from the manufacturer database, will be the subject of future studies.

## Conclusion

In REMS scans from Caucasian women, demographic and anthropometric variables accounted for only part of REMS BMD under statistically reliable independent testing. Using age and weight alone, approximately 30% of femoral-neck REMS-BMD variance and more than 40% of total-hip and lumbar-spine REMS-BMD variance remained unexplained. Additional sensitivity analyses using alternative anthropometric models yielded similar findings: roughly one third of total-hip and lumbar-spine REMS-BMD variance and one fourth of femoral-neck REMS-BMD variance were always left unexplained by anthropometric inputs. Very high R^2^ values (above 0.76) were observed only within training data, whereas that explainability dropped consistently in independent test sets. BMI-containing, penalized, and spline-based sensitivity analyses did not change this conclusion. These findings do not deny the expected relevant association between REMS BMD and demographic/anthropometric variables, but indicate that such association does not fully determine REMS BMD and that the apparent anthropometric explainability of REMS BMD is strongly sample-dependent and substantially overstated by same-cohort analyses with respect to independent testing.

## Supplementary Information

Below is the link to the electronic supplementary material.

Supplementary Material 1

Supplementary Material 2

## Data Availability

De-identified aggregated outputs underlying the present analyses are available from the corresponding author on reasonable request.
