# Supplementary material for: Methodological Clarification and Analysis of Demographic and Anthropometric Determinants in the Calculation of REMS Bone Mineral Density
Source: Calcif Tissue Int. 2026 May 19;117(1):85. doi: 10.1007/s00223-026-01547-1 (PMC13186884; doi:10.1007/s00223-026-01547-1)
Supplement: Supplementary file 1 — Supplementary Material 1 [file 223_2026_1547_MOESM1_ESM.docx]

# Supplementary Material

This supplement reports additional balance diagnostics and exploratory robustness analyses referred to in the paper main text. Absolute standardized mean differences (|SMD|) close to 0 and overlap coefficients derived from kernel-density estimation (OVL-KDE) close to 1 indicate strong practical overlap. Adjusted R² is shown only where a single fixed predictor count is directly interpretable. Variance inflation factors (VIFs) were used only for the age + weight + BMI model to quantify how much weight and BMI partly restated the same body-size information.

# Table S1. Development-versus-test balance diagnostics: |SMD| and OVL-KDE.

| **Cohort** | **Variable** | **\|SMD\|** | **OVL-KDE** |
| --- | --- | --- | --- |
| Lumbar spine | age | 0.001 | 0.972 |
| Lumbar spine | bmd_total | 0.013 | 0.964 |
| Lumbar spine | bmi | 0.010 | 0.986 |
| Lumbar spine | height | 0.002 | 0.982 |
| Lumbar spine | weight | 0.008 | 0.991 |
| Proximal femur | age | 0.010 | 0.983 |
| Proximal femur | bmd_fn | 0.018 | 0.968 |
| Proximal femur | bmd_th | 0.018 | 0.961 |
| Proximal femur | bmi | 0.013 | 0.971 |
| Proximal femur | height | 0.005 | 0.945 |
| Proximal femur | weight | 0.025 | 0.955 |

# Table S2. Comparative model performance at train size 400, including adjusted R^2^ for unpenalized models.

For each site and model family, reported values are medians across 100 repeated random training extractions with train size 400. Entries are shown as ordinary R² / adjusted R² where applicable; for ridge- and lasso-penalized models and for the spline-based model, adjusted R² is not shown because a single fixed predictor count is not directly interpretable.

## Lumbar spine

| **Model family** | **Train R² / adj.R²** | **Test R² / adj.R²** | **Median gap [min-max]** |
| --- | --- | --- | --- |
| Primary (age + weight) | 0.77 / 0.77 | 0.58 / 0.58 | 0.18 [0.10-0.25] |
| Sensitivity 1 (age + BMI) | 0.54 / 0.54 | 0.39 / 0.39 | 0.16 [0.02-0.25] |
| Sensitivity 2 (age + weight + BMI) | 0.81 / 0.80 | 0.64 / 0.64 | 0.17 [0.12-0.21] |
| Hypothetical maximum-explainability | 0.84 / 0.83 | 0.66 / 0.66 | 0.17 [0.12-0.22] |
| Ridge-penalized hypothetical | 0.83 / — | 0.66 / — | 0.17 [0.12-0.22] |
| Lasso-penalized hypothetical | 0.83 / — | 0.66 / — | 0.17 [0.12-0.23] |
| Spline-based model | 0.86 / — | 0.68 / — | 0.18 [0.12-0.23] |

## Total hip

| **Model family** | **Train R² / adj.R²** | **Test R² / adj.R²** | **Median gap [min-max]** |
| --- | --- | --- | --- |
| Primary (age + weight) | 0.83 / 0.83 | 0.57 / 0.57 | 0.26 [0.22-0.30] |
| Sensitivity 1 (age + BMI) | 0.84 / 0.84 | 0.71 / 0.71 | 0.13 [0.10-0.17] |
| Sensitivity 2 (age + weight + BMI) | 0.87 / 0.87 | 0.68 / 0.68 | 0.19 [0.16-0.22] |
| Hypothetical maximum-explainability | 0.89 / 0.89 | 0.69 / 0.69 | 0.20 [0.17-0.23] |
| Ridge-penalized hypothetical | 0.89 / — | 0.69 / — | 0.20 [0.17-0.22] |
| Lasso-penalized hypothetical | 0.89 / — | 0.69 / — | 0.20 [0.17-0.22] |
| Spline-based model | 0.90 / — | 0.70 / — | 0.20 [0.17-0.32] |

## Femoral neck

| **Model family** | **Train R² / adj.R²** | **Test R² / adj.R²** | **Median gap [min-max]** |
| --- | --- | --- | --- |
| Primary (age + weight) | 0.93 / 0.93 | 0.70 / 0.70 | 0.23 [0.21-0.25] |
| Sensitivity 1 (age + BMI) | 0.85 / 0.85 | 0.74 / 0.74 | 0.11 [0.08-0.15] |
| Sensitivity 2 (age + weight + BMI) | 0.94 / 0.94 | 0.75 / 0.75 | 0.20 [0.18-0.21] |
| Hypothetical maximum-explainability | 0.95 / 0.95 | 0.75 / 0.75 | 0.20 [0.19-0.22] |
| Ridge-penalized hypothetical | 0.95 / — | 0.75 / — | 0.20 [0.19-0.22] |
| Lasso-penalized hypothetical | 0.95 / — | 0.75 / — | 0.20 [0.19-0.22] |
| Spline-based model | 0.95 / — | 0.75 / — | 0.20 [0.19-0.22] |

# Table S3. VIFs for the supplementary age + weight + BMI model.

| **Site** | **Predictor** | **VIF** | **Redundancy** |
| --- | --- | --- | --- |
| Lumbar spine | age | 1.12 | low |
| Lumbar spine | weight | 2.99 | moderate |
| Lumbar spine | bmi | 3.06 | moderate |
| Proximal femur | age | 1.11 | low |
| Proximal femur | weight | 3.51 | moderate |
| Proximal femur | bmi | 3.58 | moderate |

# Table S4. Paired Wilcoxon signed-rank tests comparing train R² and fixed internal-test R² across the 100 repeated runs at train size 400.

| **Model family** | **Lumbar spine** | **Total hip** | **Femoral neck** |
| --- | --- | --- | --- |
| Primary anthropometric model (age + weight) | p < 0.001 | p < 0.001 | p < 0.001 |
| Sensitivity 1 (age + BMI) | p < 0.001 | p < 0.001 | p < 0.001 |
| Sensitivity 2 (age + weight + BMI) | p < 0.001 | p < 0.001 | p < 0.001 |
| Hypothetical maximum-explainability expanded model | p < 0.001 | p < 0.001 | p < 0.001 |
| Ridge-penalized expanded model | p < 0.001 | p < 0.001 | p < 0.001 |
| Lasso-penalized expanded model | p < 0.001 | p < 0.001 | p < 0.001 |
| Spline-based model | p < 0.001 | p < 0.001 | p < 0.001 |

Note. Tests were performed separately for each site and model family. In all comparisons, train R² exceeded fixed internal-test R² in 100/100 repeated runs; the Wilcoxon signed-rank statistic was W = 0 in all comparisons.

# Table S5. Distribution of train and fixed-test R² across 100 repeated random training extractions for all model families.

| **Site** | **Model family** | **Train size** | **Train R² median [P10-P90]** | **Fixed-test R² median [P10-P90]** |
| --- | --- | --- | --- | --- |
| Lumbar spine | Primary (age + weight) | 100 | 0.78 [0.72-0.82] | 0.58 [0.57-0.59] |
| Lumbar spine | Primary (age + weight) | 200 | 0.77 [0.72-0.81] | 0.58 [0.57-0.59] |
| Lumbar spine | Primary (age + weight) | 300 | 0.77 [0.73-0.80] | 0.58 [0.58-0.59] |
| Lumbar spine | Primary (age + weight) | 400 | 0.77 [0.74-0.81] | 0.58 [0.58-0.59] |
| Lumbar spine | Sensitivity 1 (age + BMI) | 100 | 0.54 [0.39-0.62] | 0.38 [0.35-0.39] |
| Lumbar spine | Sensitivity 1 (age + BMI) | 200 | 0.55 [0.46-0.61] | 0.38 [0.37-0.39] |
| Lumbar spine | Sensitivity 1 (age + BMI) | 300 | 0.54 [0.48-0.59] | 0.39 [0.38-0.39] |
| Lumbar spine | Sensitivity 1 (age + BMI) | 400 | 0.54 [0.49-0.59] | 0.39 [0.38-0.39] |
| Lumbar spine | Sensitivity 2 (age + weight + BMI) | 100 | 0.82 [0.75-0.86] | 0.63 [0.61-0.64] |
| Lumbar spine | Sensitivity 2 (age + weight + BMI) | 200 | 0.81 [0.76-0.85] | 0.63 [0.62-0.64] |
| Lumbar spine | Sensitivity 2 (age + weight + BMI) | 300 | 0.81 [0.78-0.84] | 0.64 [0.63-0.64] |
| Lumbar spine | Sensitivity 2 (age + weight + BMI) | 400 | 0.81 [0.78-0.83] | 0.64 [0.63-0.64] |
| Lumbar spine | Hypothetical maximum-explainability | 100 | 0.87 [0.81-0.90] | 0.64 [0.60-0.66] |
| Lumbar spine | Hypothetical maximum-explainability | 200 | 0.84 [0.80-0.87] | 0.66 [0.64-0.67] |
| Lumbar spine | Hypothetical maximum-explainability | 300 | 0.84 [0.81-0.87] | 0.66 [0.65-0.67] |
| Lumbar spine | Hypothetical maximum-explainability | 400 | 0.84 [0.81-0.86] | 0.66 [0.66-0.67] |
| Lumbar spine | Ridge-penalized hypothetical | 100 | 0.85 [0.79-0.89] | 0.65 [0.63-0.66] |
| Lumbar spine | Ridge-penalized hypothetical | 200 | 0.84 [0.80-0.87] | 0.66 [0.65-0.66] |
| Lumbar spine | Ridge-penalized hypothetical | 300 | 0.84 [0.81-0.86] | 0.66 [0.65-0.67] |
| Lumbar spine | Ridge-penalized hypothetical | 400 | 0.83 [0.81-0.86] | 0.66 [0.66-0.67] |
| Lumbar spine | Lasso-penalized hypothetical | 100 | 0.85 [0.80-0.89] | 0.65 [0.63-0.66] |
| Lumbar spine | Lasso-penalized hypothetical | 200 | 0.84 [0.80-0.87] | 0.66 [0.65-0.67] |
| Lumbar spine | Lasso-penalized hypothetical | 300 | 0.83 [0.80-0.86] | 0.66 [0.65-0.67] |
| Lumbar spine | Lasso-penalized hypothetical | 400 | 0.83 [0.81-0.86] | 0.66 [0.65-0.67] |
| Lumbar spine | Spline-based model | 100 | 0.88 [0.84-0.92] | 0.65 [0.61-0.67] |
| Lumbar spine | Spline-based model | 200 | 0.87 [0.84-0.90] | 0.67 [0.65-0.69] |
| Lumbar spine | Spline-based model | 300 | 0.86 [0.83-0.89] | 0.68 [0.66-0.69] |
| Lumbar spine | Spline-based model | 400 | 0.86 [0.84-0.88] | 0.68 [0.67-0.70] |
| Total hip | Primary (age + weight) | 100 | 0.82 [0.79-0.86] | 0.57 [0.55-0.57] |
| Total hip | Primary (age + weight) | 200 | 0.83 [0.80-0.85] | 0.57 [0.56-0.57] |
| Total hip | Primary (age + weight) | 300 | 0.83 [0.81-0.84] | 0.57 [0.56-0.57] |
| Total hip | Primary (age + weight) | 400 | 0.83 [0.81-0.84] | 0.57 [0.56-0.57] |
| Total hip | Sensitivity 1 (age + BMI) | 100 | 0.84 [0.80-0.87] | 0.70 [0.69-0.71] |
| Total hip | Sensitivity 1 (age + BMI) | 200 | 0.84 [0.81-0.87] | 0.70 [0.70-0.71] |
| Total hip | Sensitivity 1 (age + BMI) | 300 | 0.84 [0.82-0.86] | 0.70 [0.70-0.71] |
| Total hip | Sensitivity 1 (age + BMI) | 400 | 0.84 [0.81-0.85] | 0.71 [0.70-0.71] |
| Total hip | Sensitivity 2 (age + weight + BMI) | 100 | 0.88 [0.85-0.90] | 0.68 [0.66-0.69] |
| Total hip | Sensitivity 2 (age + weight + BMI) | 200 | 0.88 [0.86-0.89] | 0.68 [0.67-0.69] |
| Total hip | Sensitivity 2 (age + weight + BMI) | 300 | 0.87 [0.86-0.89] | 0.68 [0.67-0.69] |
| Total hip | Sensitivity 2 (age + weight + BMI) | 400 | 0.87 [0.85-0.88] | 0.68 [0.67-0.69] |
| Total hip | Hypothetical maximum-explainability | 100 | 0.90 [0.87-0.92] | 0.68 [0.66-0.70] |
| Total hip | Hypothetical maximum-explainability | 200 | 0.90 [0.88-0.91] | 0.69 [0.67-0.70] |
| Total hip | Hypothetical maximum-explainability | 300 | 0.89 [0.87-0.91] | 0.69 [0.68-0.70] |
| Total hip | Hypothetical maximum-explainability | 400 | 0.89 [0.88-0.90] | 0.69 [0.69-0.70] |
| Total hip | Ridge-penalized hypothetical | 100 | 0.90 [0.88-0.92] | 0.69 [0.66-0.70] |
| Total hip | Ridge-penalized hypothetical | 200 | 0.89 [0.88-0.91] | 0.69 [0.68-0.70] |
| Total hip | Ridge-penalized hypothetical | 300 | 0.89 [0.88-0.91] | 0.69 [0.68-0.70] |
| Total hip | Ridge-penalized hypothetical | 400 | 0.89 [0.88-0.90] | 0.69 [0.69-0.70] |
| Total hip | Lasso-penalized hypothetical | 100 | 0.90 [0.88-0.92] | 0.68 [0.66-0.70] |
| Total hip | Lasso-penalized hypothetical | 200 | 0.89 [0.87-0.91] | 0.69 [0.67-0.70] |
| Total hip | Lasso-penalized hypothetical | 300 | 0.89 [0.88-0.90] | 0.69 [0.68-0.70] |
| Total hip | Lasso-penalized hypothetical | 400 | 0.89 [0.88-0.90] | 0.69 [0.68-0.70] |
| Total hip | Spline-based model | 100 | 0.91 [0.89-0.93] | 0.67 [0.63-0.69] |
| Total hip | Spline-based model | 200 | 0.90 [0.89-0.92] | 0.69 [0.68-0.71] |
| Total hip | Spline-based model | 300 | 0.90 [0.89-0.91] | 0.70 [0.69-0.71] |
| Total hip | Spline-based model | 400 | 0.90 [0.89-0.91] | 0.70 [0.69-0.71] |
| Femoral neck | Primary (age + weight) | 100 | 0.93 [0.91-0.95] | 0.69 [0.69-0.70] |
| Femoral neck | Primary (age + weight) | 200 | 0.93 [0.92-0.94] | 0.70 [0.69-0.70] |
| Femoral neck | Primary (age + weight) | 300 | 0.93 [0.92-0.94] | 0.70 [0.69-0.70] |
| Femoral neck | Primary (age + weight) | 400 | 0.93 [0.92-0.94] | 0.70 [0.69-0.70] |
| Femoral neck | Sensitivity 1 (age + BMI) | 100 | 0.86 [0.82-0.89] | 0.73 [0.73-0.74] |
| Femoral neck | Sensitivity 1 (age + BMI) | 200 | 0.85 [0.83-0.88] | 0.74 [0.73-0.74] |
| Femoral neck | Sensitivity 1 (age + BMI) | 300 | 0.85 [0.83-0.87] | 0.74 [0.73-0.74] |
| Femoral neck | Sensitivity 1 (age + BMI) | 400 | 0.85 [0.83-0.86] | 0.74 [0.73-0.74] |
| Femoral neck | Sensitivity 2 (age + weight + BMI) | 100 | 0.95 [0.93-0.96] | 0.75 [0.74-0.75] |
| Femoral neck | Sensitivity 2 (age + weight + BMI) | 200 | 0.94 [0.94-0.95] | 0.75 [0.74-0.75] |
| Femoral neck | Sensitivity 2 (age + weight + BMI) | 300 | 0.94 [0.94-0.95] | 0.75 [0.74-0.75] |
| Femoral neck | Sensitivity 2 (age + weight + BMI) | 400 | 0.94 [0.94-0.95] | 0.75 [0.74-0.75] |
| Femoral neck | Hypothetical maximum-explainability | 100 | 0.95 [0.94-0.96] | 0.74 [0.72-0.75] |
| Femoral neck | Hypothetical maximum-explainability | 200 | 0.95 [0.94-0.96] | 0.74 [0.74-0.75] |
| Femoral neck | Hypothetical maximum-explainability | 300 | 0.95 [0.94-0.95] | 0.75 [0.74-0.75] |
| Femoral neck | Hypothetical maximum-explainability | 400 | 0.95 [0.94-0.95] | 0.75 [0.74-0.75] |
| Femoral neck | Ridge-penalized hypothetical | 100 | 0.95 [0.94-0.96] | 0.74 [0.72-0.75] |
| Femoral neck | Ridge-penalized hypothetical | 200 | 0.95 [0.94-0.96] | 0.75 [0.74-0.75] |
| Femoral neck | Ridge-penalized hypothetical | 300 | 0.95 [0.94-0.96] | 0.75 [0.74-0.75] |
| Femoral neck | Ridge-penalized hypothetical | 400 | 0.95 [0.94-0.95] | 0.75 [0.74-0.75] |
| Femoral neck | Lasso-penalized hypothetical | 100 | 0.95 [0.94-0.96] | 0.74 [0.73-0.75] |
| Femoral neck | Lasso-penalized hypothetical | 200 | 0.95 [0.94-0.96] | 0.74 [0.73-0.75] |
| Femoral neck | Lasso-penalized hypothetical | 300 | 0.95 [0.94-0.96] | 0.75 [0.74-0.75] |
| Femoral neck | Lasso-penalized hypothetical | 400 | 0.95 [0.94-0.95] | 0.75 [0.74-0.75] |
| Femoral neck | Spline-based model | 100 | 0.96 [0.95-0.97] | 0.73 [0.72-0.75] |
| Femoral neck | Spline-based model | 200 | 0.95 [0.95-0.96] | 0.74 [0.73-0.75] |
| Femoral neck | Spline-based model | 300 | 0.95 [0.94-0.96] | 0.75 [0.74-0.75] |
| Femoral neck | Spline-based model | 400 | 0.95 [0.95-0.96] | 0.75 [0.74-0.75] |
