# Supplementary material for: Methodological Clarification and Analysis of Demographic and Anthropometric Determinants in the Calculation of REMS Bone Mineral Density
Source: Calcif Tissue Int. 2026 May 19;117(1):85. doi: 10.1007/s00223-026-01547-1 (PMC13186884; doi:10.1007/s00223-026-01547-1)
Supplement: Supplementary file 2 — Supplementary Material 2 [file 223_2026_1547_MOESM2_ESM.pdf]

CONSENSO INFORMATO

Titolo studio	[redacted]

Io sottoscritto …………………………………… ……………………………..………

Cognome nome

età (anni) ………………… data di nascita ……. / ……. / …….

Indirizzo …………………………………………………………..……… …………………

P.zza / Via / V.le Numero Civico

Città ………………..……………………… telefono …………………………..

dichiaro di:

- partecipare volontariamente allo studio di cui mi sono stati spiegati e di cui ho compreso lo scopo, le procedure alle quali potrò essere esposto, i possibili rischi e i benefici e le possibili alternative
- aver preso visione delle "Informazioni scritte per il paziente" facenti parte di questo consenso, che confermano quanto mi è stato detto sullo studio
- aver avuto l'opportunità di porre domande chiarificatrici e di aver avuto risposte soddisfacenti
- aver avuto tutto il tempo necessario prima di decidere se partecipare o meno
- non aver avuto alcuna coercizione indebita nella richiesta del Consenso
- acconsentire/non acconsentire a che il mio medico curante venga informato dallo sperimentatore circa la mia partecipazione allo studio sperimentale in oggetto
- ai sensi del Decreto legislativo 30.6.03 n. 196 codice privacy, autorizzo il proponente di questo studio, a sottoporre a trattamento (nel senso specificato dalla legge) i dati personali e sensibili che mi riguardano, forniti allo sperimentatore, in quanto necessari alla mia partecipazione allo studio in oggetto.

Data ………. / ………. / ……….

Firma del paziente (adulto, minore maturo) ....

Firma di entrambi genitori in caso di soggetto minore ....

....

Firma del rappresentante legale (in caso di paziente inabilitato, interdetto o con amministratore di sostegno) ....

Io sottoscritto PROF. [redacted] [redacted]

Cognome Nome

Dichiaro che il paziente ha firmato spontaneamente la sua partecipazione allo studio

Dichiaro inoltre di:

- aver fornito al paziente esaurienti spiegazioni in merito alle finalità dello studio, alle procedure, ai possibili rischi e benefici e alle possibili alternative;
- aver verificato che il paziente abbia sufficientemente compreso le informazioni fornitegli
- aver lasciato al paziente il tempo necessario e la possibilità di fare domande in merito allo studio
- non aver esercitato alcuna coercizione od influenza indebita nella richiesta del Consenso

Data ………. / ………. / ……….

....

Firma del Medico che ha informato il paziente
e richiesto il consenso informato

NOTA BENE

una copia del presente modulo, firmato e datato, allegato alle "Informazioni Scritte per il Paziente" è consegnata al Paziente stesso

INFORMATIVA PER IL PAZIENTE

[redacted]

PROMOTORE: [redacted]

Gentile Signora / Egregio Signore,

Le è stato chiesto di partecipare ad uno studio clinico sperimentale e questo documento ha lo scopo di informarLa sulla natura dello studio, sul fine che esso si propone, su ciò che comporterà per Lei una tale partecipazione, sui suoi diritti e le sue responsabilità.

La prego di leggere attentamente queste informazioni scritte prima di prendere una decisione in merito ad una eventuale Sua partecipazione allo studio. Lei avrà a disposizione tutto il tempo necessario per decidere se partecipare o meno.

Potrà, inoltre, porre liberamente qualsiasi domanda di chiarimento e riproporre ogni quesito che non abbia ricevuto una risposta chiara ed esauriente.

Nel caso in cui, dopo aver letto e compreso tutte le informazioni ivi fornite, decidesse di voler partecipare allo studio clinico, Le chiederò di voler firmare e personalmente datare il modulo di Consenso Informato allegato a questo documento.

CHE COSA SI PROPONE LO STUDIO

Lo studio, proposto dal [redacted] ha l'obiettivo di studiare ecograficamente lo stato delle ossa senza che ciò comporti alcun rischio aggiuntivo.

COSA COMPORTA LA SUA PARTECIPAZIONE ALLO STUDIO

Nel caso in cui Lei decidesse di partecipare allo studio, La informiamo che, dopo aver valutato la possibilità di poterLa includere nella ricerca e dopo aver effettuato tutti i trattamenti medici/strumentali per Lei previsti indipendentemente dalla Sua partecipazione o meno a questa ricerca, lo studio prevede che lei si sottoponga ad un semplice esame ecografico esterno, che permetterà al Gruppo di Ricerca (bioingegneri, biologi, medici) di studiare nuove metodologie di valutazioni della struttura ossea, e, in un prossimo futuro, adottare un protocollo diagnostico che non richieda l'uso di radiazioni ionizzanti (es. raggi X).

La informiamo che, essendo questo studio in fase iniziale, la valutazione delle immagini non sarà usata per la formulazione della sua diagnosi, ma tali immagini saranno archiviate in forma anonima e studiate successivamente e/o retrospettivamente nel rispetto delle norme e leggi correnti.

La informiamo, inoltre, che la partecipazione alla ricerca non comporta per Lei alcun aggravio di spese che saranno tutte a carico del promotore.

Non ci sono rischi derivanti dalla partecipazione allo studio in quanto vengono impiegate strumentazioni non invasive, non ionizzanti e senza alcuna interazione biologica con il paziente.

INFORMAZIONI CIRCA I RISULTATI DELLO STUDIO

Se Lei lo richiederà, alla fine dello studio potranno esserle comunicati i risultati dello studio ed in particolare quelli che La riguardano.

ULTERIORI INFORMAZIONI

Per ulteriori informazioni e comunicazioni durante lo studio sarà a disposizione il seguente personale:

- Prof. [redacted]

Il protocollo dello studio che Le è stato proposto è stato approvato dal Comitato Etico che ha tra l'altro verificato la conformità alle Norme di Buona Pratica Clinica della Unione Europea ed in accordo ai principi etici espressi nelle Dichiarazione di Helsinki ed è stato approvato dal Comitato Etico di questa struttura.

Lei potrà segnalare qualsiasi fatto ritenga opportuno evidenziare, relativamente alla ricerca che La riguarda, al Comitato Etico e/o alla Direzione Sanitaria di questa struttura ospedaliera.

INFORMATIVA E MANIFESTAZIONE DEL CONSENSO AL TRATTAMENTO DEI DATI PERSONALI

Finalità del trattamento. Titolare e responsabile del trattamento

- Il Reparto di [redacted]

, quale Centro di sperimentazione, e il Promotore [redacted] che ha commissionato lo studio [redacted] che Le è stato presentato, utilizzeranno i Suoi dati personali, quali autonomi Titolari del trattamento, in accordo alle responsabilità previste dalle norme di buona pratica clinica (d.l. 211/2003) e dalle disposizioni in materia di protezione dei dati personali (d.l. 196/2003).

Il Prof. [redacted] che segue lo studio presso il Centro di sperimentazione, è individuato quale sperimentatore principale e dunque quale responsabile del trattamento dei dati in riferimento alla titolarità del Centro di sperimentazione stesso.

Il [redacted], in quanto ente promotore, gestirà internamente le attività di archiviazione e analisi dati in forma anonima. Lo Sperimentatore principale si avvarrà della collaborazione del personale del [redacted], individuato quale incaricato del trattamento. Tali soggetti sono individuati dal promotore quali responsabili esterni del trattamento dei dati in riferimento alla titolarità del promotore stesso.

Conferimento dei dati

Il trattamento dei dati personali relativi alla sua condizione clinica è indispensabile allo svolgimento dello studio: il rifiuto di conferirli non Le consentirà di parteciparvi.

Tipologia e natura dei dati

Nel corso dello studio verranno trattate le seguenti tipologie di dati: età, altezza e peso del paziente, informazioni relative alla origine, agli stili di vita, acquisiti mediante colloquio con l'interessato stesso unitamente all'esito dell'esame DXA.

Modalità del trattamento

I dati saranno trattati mediante strumenti elettronici protetti.
Il personale medico e paramedico (sperimentatori) che La seguiranno nello studio La identificheranno con un codice: i dati che La riguardano, raccolti nel corso dello studio saranno registrati, elaborati e conservati unitamente a tale codice. Soltanto i soggetti autorizzati potranno collegare questo codice al Suo nominativo.
La Sua partecipazione allo studio implica che, in conformità alla normativa sulle sperimentazioni cliniche, il personale che esegue il monitoraggio e la verifica dello studio, il Comitato etico e le autorità sanitarie potranno conoscere i dati (anche identificativi) che La riguardano, eventualmente compresi quelli contenuti nella Sua documentazione clinica originale.
I dati potranno essere diffusi, ad esempio attraverso pubblicazioni scientifiche, statistiche e convegni scientifici, solo in forma rigorosamente anonima.

Esercizio dei diritti

Potrà interrompere in ogni momento e senza fornire alcuna giustificazione la Sua partecipazione allo studio; in conseguenza di ciò, non saranno raccolti ulteriori dati che La riguardano.

Consenso

Sottoscrivendo tale modulo acconsento al trattamento dei miei dati personali per gli scopi della ricerca nei limiti e con le modalità indicate nell'informativa fornitami con il presente documento.

Nome e Cognome dell'interessato ________________________________
(in stampatello)

Firma dell'interessato ________________________________

Data ______________________

Da sottoporre agli interessati unitamente al modulo di consenso informato che descrive le caratteristiche scientifiche dello studio, anche mediante integrazione dello stesso
